# Pediatric tropical medicine: The neglected diseases of children

**DOI:** 10.1371/journal.pntd.0007008

**Published:** 2019-05-09

**Authors:** Peter J. Hotez, Audrey R. Odom John, A. Desiree LaBeaud

**Affiliations:** 1 Texas Children’s Hospital Center for Vaccine Development, Departments of Pediatrics and Molecular Virology and Microbiology, National School of Tropical Medicine, Baylor College of Medicine, Houston, Texas, United States of America; 2 Department of Biology, Baylor University, Waco, Texas, United States of America; 3 Departments of Pediatrics and Molecular Microbiology, Washington University School of Medicine, St. Louis, Missouri, United States of America; 4 Department of Pediatrics, Division of Infectious Diseases, Stanford University, Stanford, California, United States of America; Weill Cornell Medical College, UNITED STATES

## Introduction

New information released by the Infectious Diseases Society of America (IDSA) and the National Resident Matching Program shows continued declines in interest in pediatric infectious diseases as a career, highlighting that we risk losing a generation of trained experts in pediatric infectious and tropical diseases. The loss paradoxically coincides with updated estimates revealing the disproportionate global health impact of tropical infectious diseases on children. Our objective is to highlight several ominous trends, including our findings that (1) the pediatric tropical disease burdens are huge and do not appear to be declining and (2) fewer trainees are entering the field. Included here are are some key suggestions to address these concerns.

## Pediatric disease burden

It is often underappreciated how the world’s tropical infections, especially malaria and the neglected tropical diseases (NTDs), disproportionately affect children. The latest Global Burden of Disease Study (GBD) from 2017 provides some chilling estimates [1–3]. Together, malaria and NTDs cause an estimated 720,100 deaths and 62 million disability-adjusted life years (DALYs), ranking these conditions among our leading global health threats [1–3]. But the GBD 2017 numbers also highlight the fact that approximately one-half of those deaths and DALYs affect children under the age of 5 years, whereas approximately two-thirds of deaths and DALYs affect children and adolescents under the age of 20 years [4]. The bottom line is that much of what we know as the field of tropical medicine is very much in essence the field of tropical pediatrics.

Shown in Tables 1 and 2 are the rankings of the most common tropical infectious diseases of children in terms of their prevalence or incidence.

**Table 1:** Prevalence or incidence of tropical infections in children under 5 years old (information from [1–4]).

Disease	Prevalence or *Incidence*^a^	Precentage of global DALYs in children under 5 years old
Malaria	*77*.*0 million*	69%
Ascariasis	45.3 million	32%
Hookworm infection	16.7 million	07%
Trichuriasis	13.4 million	03%
Dengue	*7*.*2 million*	23%

^a^Incidence data in italicized type; prevalence data in nonitalicized type.

Abbreviation: DALY, disability-adjusted life year.

**Table 2:** Prevalence or incidence of tropical infections in children and adolescents under 20 years old (information from [1–4]).

Disease	Prevalence or *Incidence*^a^	Percentage of global DALYs in children and adolescents under 20 years old
Ascariasis	221.9 million	66%
Malaria	*155*.*3 million*	82%
Trichuriasis	109.1 million	38%
Hookworm disease	93.3 million	41%
Dengue	*43*.*0 million*	50%

^a^Incidence data in italicized type; prevalence data in nonitalicized type.

Abbreviation: DALY, disability-adjusted life year.

For children under the age of 5 years, the most ubiquitous tropical infection is malaria, followed by the three major soil-transmitted helminth infections (ascariasis, trichuriasis, and hookworm infection) and dengue. For children and adolescents (under age 20 years), the major tropical infections are mostly the same, although for this age group, schistosomiasis is also an important NTD.

What does this information mean for the bigger global health picture? First, it is a critical reminder of the disproportionate impact of NTDs on children and adolescents. Not only are children more likely to be infected in the first place but also pediatric infections are often more severe. Even non-life-threatening infections in children may cause profound life-long impacts on growth and neurodevelopment, with invisible health, economic, and social consequences. For example, malaria is a major cause of death in young children, in Africa and elsewhere, and frequently combines with hookworm to result in profound and incapacitating anemia [5]. Similarly, diarrheal disease due to cryptosporidiosis has both short-term mortality risks and long-term impacts on weight gain and growth [6], even though it is not included in WHO’s list of NTDs. These diseases are also major afflictions of adolescents, especially for adolescent girls, in whom these tropical infections lead not only to malnutrition and anemia but also to genitourinary tract disease, which may promote the spread of HIV [7, 8]. In addition, as adolescent girls reach reproductive age, many of these infections, such as malaria and dengue, have harmful effects on gestation and birth, leading to poor pregnancy, birth, and early-childhood outcomes that perpetuate the cycle of poverty, disease, and inequity [9–13]. Lastly, this information also has important implications for training, workforce development, and research (as outlined in detail below), both for resource-poor countries in Africa, Asia, and Latin America and for the NTDs noted to occur in areas of deep poverty of the United States and other high-income nations.

## Training

The impact of tropical infections on children is an important message for workforce training. In the US, recent information from the IDSA reveals that many fellowship training slots in pediatric infectious diseases currently go unfilled [14]. For example, in 2015, almost one-half of the 60–70 available fellowship slots in pediatric infectious diseases (including those recognized by the Pediatric Infectious Diseases Society [PIDS]) went unmatched [14]. In this past year (2018 match), 44% of 72 slots remained unfilled, signaling an ongoing downward trend that is likely to continue [15]. Among the reasons for this situation are the long work hours, lack of job opportunities in desired locations [16], and comparatively low compensation, compounded by rising educational debt and increased cost of living. Overall, in the US, we are simply not training sufficient numbers of future clinicians, researchers, and leaders who are knowledgeable about pediatric tropical infectious diseases.

Outside of the US, we have less information about pediatric tropical medicine workforce capacity. A report in 2011 found that there were fewer than 24 physicians with formal training in infectious diseases in the nation of India, with most of those individuals obtaining their training in the US, United Kingdom, or Australia [17]. Similarly, it has been estimated that the number of pediatricians practicing on the African continent is in the range of 0.03 to 0.8 per 100,000 population, compared with 11 to 86 per 100,000 population in some European countries (roughly a 100-fold differential) [18].

Luckily, there are some important efforts underway to address gaps in the global pediatric tropical disease workforce [19]. An African Paediatric Fellowship Programme has been shaped at the University of Cape Town in South Africa, in which trainees spend between 6 months and 2 years in pediatric training before they return to their home institutions [18], and the Pediatric AIDS Corps (and its successor, the Texas Children’s Global Health Corps) through the Baylor College of Medicine International Pediatric AIDS Initiative (BIPAI) has trained more than 150 physicians with in-depth expertise in the management of pediatric HIV/AIDS and tropical infections since 2005, primarily for Africa [20, 21]. Currently, this initiative treats annually approximately 300,000 children in more than 12 countries [22]. The UK offers postgraduate courses in tropical pediatrics at the Liverpool School of Tropical Medicine [23], which also publishes a journal in the field, and Thailand’s Mahidol University maintains a distinguished Department of Tropical Pediatrics with a number of important offerings [24]. The American Academy of Pediatrics (AAP) maintains a section of International Child Health [25], and there are also multiple centers devoted to global child health and distinguished pediatrics departments in disease-endemic countries, although they are not necessarily specifically focused on pediatric tropical medicine.

## Research and development

Beyond the workforce, new technologies are urgently needed to address the NTDs of children worldwide. In 2016, a consensus document was prepared by the major product development partnerships for these diseases [26], but it needs to be updated and specifically focused on the needs of pediatric tropical medicine. We also need to recognize that, in the last few years, the area of global health innovation has undergone major shifts. There is now an array of new basic science approaches to neglected diseases that include not only single-cell combinatorial indexing RNA sequencing (sci-RNA-seq), gene editing, functional and comparative OMICs, and systems biology [27] but also new and alternative funding streams, which are desperately needed [28, 29]. We are also seeing shifts in the governance for the support of new diagnostics, drugs, and vaccines, some of which parallel the installation of new global health leaders [30–32]. In some cases, these activities are under threat as a result of organized antiscience movements, including a rising antivaccine initiative in the US and Europe [30].

## An expanded framework

**Fig 1:**
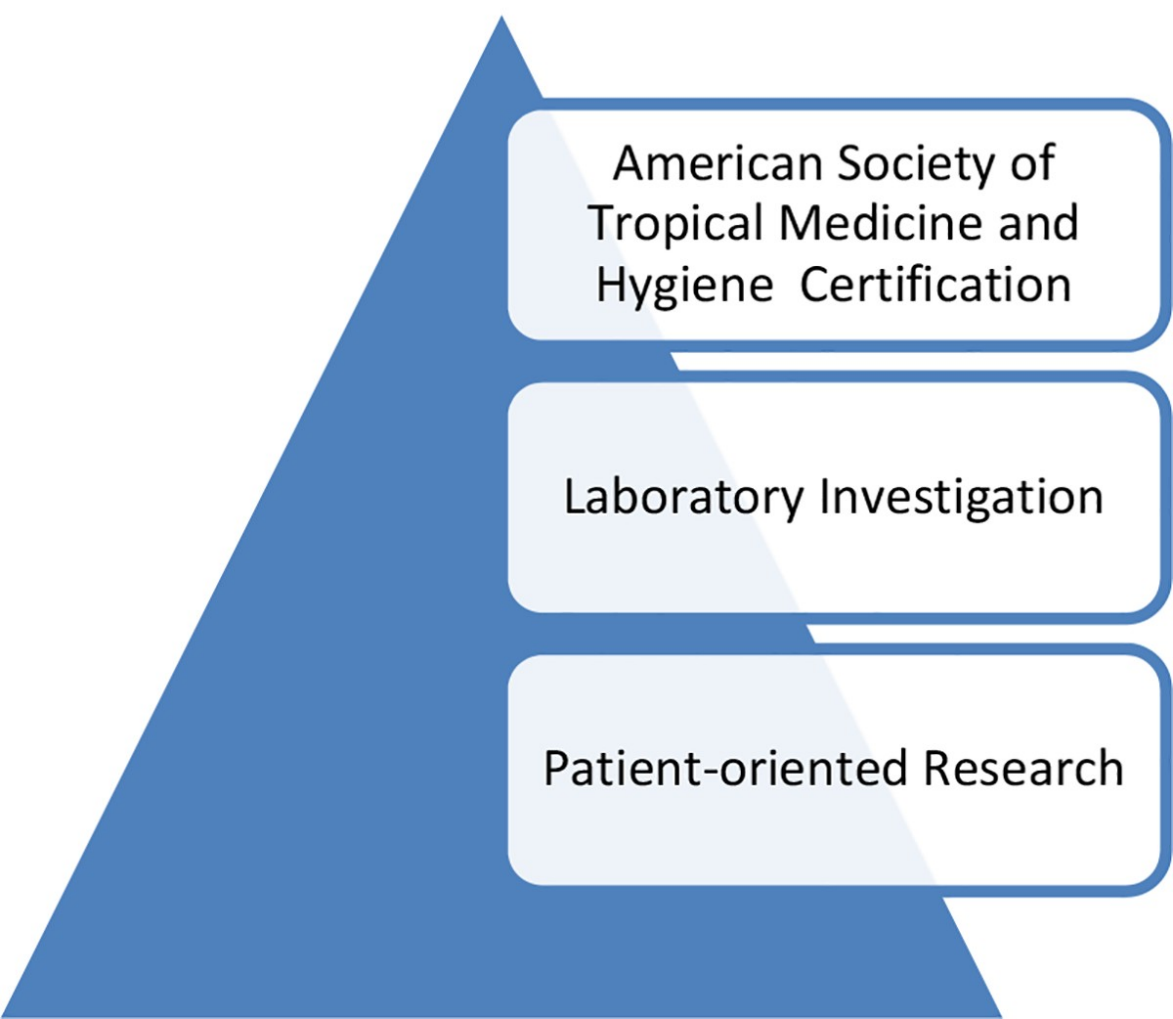
Training pathways for pediatric tropical medicine.

With increasing recognition of the severity of the tropical diseases affecting children in developing nations, as well as among the poor living in wealthy countries, including the US, Europe, and Australia [33], should we consider reassessing the overall framework of pediatric tropical medicine? In 2017, the Global Health Task Force of the American Board of Pediatrics (ABP) outlined mechanisms of how partnerships might advance global child health and health systems, with an emphasis on seven “guiding principles”—equity, sustainability, mutual benefit, humility, inclusivity, social justice, and prevention of adverse impact—and four “core practices” of communications, leadership, conflict resolution, and evaluation [34]. Under this rubric, at least one way to proceed in the US might include building on our existing pediatric infectious diseases fellowship programs and adding, as a stackable credential, one of the several diploma or certificate courses in tropical medicine (and in-country experiences) that currently lead to certification by the American Society of Tropical Medicine and Hygiene (ASTMH) [35]. This might be a first step toward new recognition and sub-subspecialty credentialing in pediatric tropical medicine, which might be paired with a period of laboratory investigative training or a partnering opportunity in disease-endemic countries for patient-oriented research (Fig 1).

More broadly, the majority of US pediatric infectious diseases training programs are based in geographic regions with a low incidence of imported tropical infectious diseases. However, NTDs can be surprisingly common [36], and imported infections are quite literally only a plane flight away. A high index of suspicion and accurate recognition of tropical infections are critical for appropriate infection control measures and global health security. To facilitate widespread awareness and improve training on pediatric-specific management of such infections, an abbreviated online training module—modeled after the highly successful primer on healthcare epidemiology and infection control currently provided by the Society for Healthcare Epidemiology of America (SHEA) [37]—could begin to fill this knowledge gap for pediatric infectious diseases trainees.

But these measures alone will not be sufficient to create a cadre of established experts in pediatric tropical medicine, nor will they advance a next generation of urgently needed drugs, diagnostics, vaccines, and vector control agents to combat these diseases. We need to not only better shape and develop career paths for our trainees but also focus attention on undergraduate medical education and provide role models for encouraging medical and graduate students to take an interest in the problem of tropical infectious diseases of children. There are a number of possible concrete strategies that would help support trainees interested in tropical pediatrics. First, financial concerns clearly impact career choices for medical graduates, who bear a median debt of approximately US$200,000 [38]. The National Institutes of Health (NIH) loan forgiveness program should be expanded to reward those individuals who spend their careers in public health or tropical infectious diseases. Second, a key concern for young physician-scientists across disciplines is the uncertainty of the research funding climate [39]. Compounding this concern are the unique challenges to research in tropical infectious diseases, including the technical complexities of basic investigations on the nonmodel organisms that cause NTDs, as well as the administrative uncertainties and capacity-building that may be required for patient-oriented research in endemic field sites. Additional NIH or foundational funding mechanisms to expand the duration of K awards or facilitate the transition from K- to R-level funding for tropical infectious diseases research could likewise alleviate concerns of trainees who may be reluctant to take on “higher-risk” scholarly activity.

Our major academic societies and organizations devoted to infectious and tropical diseases and the health of children, including IDSA, PIDS, ASTMH, AAP, and ABP, have emphasized the importance of global health training and research in the context of global security, but they might also work to launch an awareness campaign on the devastating effects of malaria, arbovirus, and helminth infections on the growth, development, and futures of children and the necessity of maintaining and supporting a pediatric tropical medicine workforce to combat these infections at home and abroad. Together, these societies have a tremendous voice and potential for impact and could help foster an environment that is conducive for enticing trainees to consider careers in pediatric tropical medicine.

Currently, the trends are ominous: (1) the pediatric tropical disease burdens are huge and do not appear to be declining, (2) fewer trainees are entering the field, and (3) the antivaccine lobby and other antiscience movements are growing and becoming more powerful. We need to begin reversing this tide and seek ways to comprehensively address pediatric tropical medicine training in the context of a global strategy for improving child health [19].
